# Effects of Infection Control Education Program Using Flipped Learning Based on the ARCS Model for Nursing Students: A Mixed Method

**DOI:** 10.3390/healthcare11202731

**Published:** 2023-10-13

**Authors:** Dain Jeong, Chang Park, Young Eun

**Affiliations:** 1 College of Nursing, Gyeongsang National University, Jinju 52727, Republic of Korea; dainj0828@gmail.com; 2 College of Nursing, University of Illinois Chicago, Chicago, IL 60612, USA; parkcg@uic.edu; 3 College of Nursing, Institute of Medical Sciences, Gyeongsang National University, Jinju 52727, Republic of Korea

**Keywords:** ARCS model, flipped learning, infection control, motivation, self-directed learning

## Abstract

This study was conducted to verify the effect of an infection control education program, using flipped learning based on the ARCS model, for nursing students. The study was a mixed-method study, composed of a nonequivalent control group, a pretest-posttest design, and focus group interviews. The total number of participants was 37, with 18 in the experimental group and 19 in the control group. The mean age of the participants was 24.32 ± 5.60 years, consisting of three males (8.1%) and thirty-four females (91.9%). The collected data were analyzed using a linear mixed-effects method. The data regarding experiences of participation were analyzed using conventional content analysis. The experimental group had a higher degree of learning motivation, self-directed learning ability, and confidence in infection control practice than the control group. In the results of the focus group interviews, the themes were derived from ‘Engaging learning experience, although difficult, in new ways of learning’. It was confirmed that the infection control education program, using flipped learning based on the ARCS model, was an effective intervention for improving nursing students’ self-directed learning ability. Therefore, it is strongly recommended to use the educational program developed in this study for infection control education for nursing students and new nurses.

## 1. Introduction

Infection prevention and control is a practical evidence-based approach aimed at preventing harm to both patients and healthcare workers by mitigating avoidable infections [1]. The importance of infection prevention and control has become more pronounced due to the emergence of novel infectious diseases, such as coronavirus disease (COVID-19) in 2020 and monkeypox (MPOX) in 2022 [2]. Nurses, who frequently interact directly with patients or perform invasive treatments, play a pivotal role in infection control [3]. To effectively manage infections, it is essential to enhance nurses’ knowledge and skills related to infection control.

In Korea, infection control education in undergraduate nursing programs encompasses both theoretical and practical components within basic and adult nursing subjects. However, it has been observed that infection control education in nursing colleges tends to emphasize theoretical content, like hand hygiene, rather than practical skills [4]. Also, in a Portuguese study [5], it was emphasized that, since infection education was not standardized, adopting an educational approach to establish a specific curriculum for infection control is crucial. As a result, nursing students often lack sufficient education in infection control for clinical settings. To address this, various instructional methods such as simulation learning [6], serious games [7], and application-based learning [8] have been introduced to nursing students for effective infection control education. Considering the occurrence of new infectious diseases like COVID-19 and MPOX, nursing students must proactively acquire infection control knowledge and skills, as they may encounter unpredictable infection situations not covered in their undergraduate courses. Therefore, providing infection control education is essential for enhancing the clinical practical skills of undergraduate nursing students [5].

Self-directed learning (SDL) ability refers to a learner’s competency to recognize learning needs, set goals, identify necessary resources and strategies, and proceed with learning [9]. As a nursing student, developing SDL ability is crucial, not only for achieving academic excellence but also for engaging in continuous learning to ensure the delivery of safe patient care [10]. Flipped learning (FL), a pedagogical approach promoting SDL ability, is actively utilized in education [11]. FL involves learners engaging in self-directed pre-class activities, using provided materials, before participating in collaborative learning activities during in-class sessions [12]. In previous studies [12], FL has been shown to result in enhancing SDL abilities. However, challenges, such as learners’ lack of motivation for pre-class activities, time constraints for pre-learning, and insufficient learning motivation during classroom and post-class activities have been reported [13]. To overcome these limitations, SDL requires motivation as a prerequisite in fostering academic curiosity [14].

Learning motivation refers to the driving force behind learners’ engagement in educational activities and plays a key role in student learning [15]; Keller [16] introduced the ARCS model to enhance learning motivation. The ARCS model emphasizes Attention (A), Relevance (R), Confidence (C), and Satisfaction (S) as key factors in motivating learners. This model fosters continual motivation and enables learners to design and manage their learning strategies to attain their educational goals [16]. The ARCS model emphasizes the importance of capturing the learner’s attention during the educational process. It encourages learners to take an active role in their own learning and activities, while also focusing on self-efficacy in acquiring new skills [17]. Additionally, a key aspect is ensuring that learners can experience satisfaction and academic achievement based on the outcomes of the learning process [17]. Motivated learners effectively utilize available learning resources and actively engage with information and events to acquire new knowledge and skills [17]. Furthermore, Keller [17] highlighted that, in educational settings, learners tend to conceal their emotions, which can lead instructors to underestimate anxiety levels. Therefore, providing learners with early success experiences to build their confidence is essential. The effects of the ARCS model in improving learning outcomes has been demonstrated through meta-analyses [18]. Previous studies applying the ARCS model have reported increased learning motivation among nursing students [19,20,21], improved SDL ability [20], increased self-efficacy, and reduced anxiety [21]. Therefore, it is imperative to evaluate the educational design and learning impact of the ARCS model in infection control education for nursing students.

Consequently, considering the diverse, complex, and unpredictable nature of contemporary infectious diseases, it is imperative that nursing students are equipped with SDL ability even after completing their undergraduate course. This will enable them to effectively handle emerging infectious diseases and infection control practices in clinical fields.

## 2. Methods

### 2.1. Study Design

This study design was mixed-method, conducted with an explanatory sequential approach. An explanatory sequential approach is a mixed method that first collects and analyses quantitative data, and then collects and analyses qualitative data to explain or generalize the quantitative data [22]. The quantitative research in this study was a quasi-experimental study design with a pretest–posttest nonequivalent control group; the qualitative research comprised focus group interviews (FGI).

### 2.2. Participants

Two nursing schools situated in different cities were selected to avoid contamination of the estimated causal effect of the infection control education program, using FL based on the ARCS model. Both colleges offer four-year nursing education programs, and it was verified that the nursing major core courses for the second and third years were identical. Furthermore, it was confirmed that neither college provided infection management education outside of its undergraduate curriculum, by the deans of each college. To avoid contamination of the intervention, participants who were G university nursing students were assigned to the experimental group, and those who were M university nursing students were assigned to the control group.

**Figure 1:**
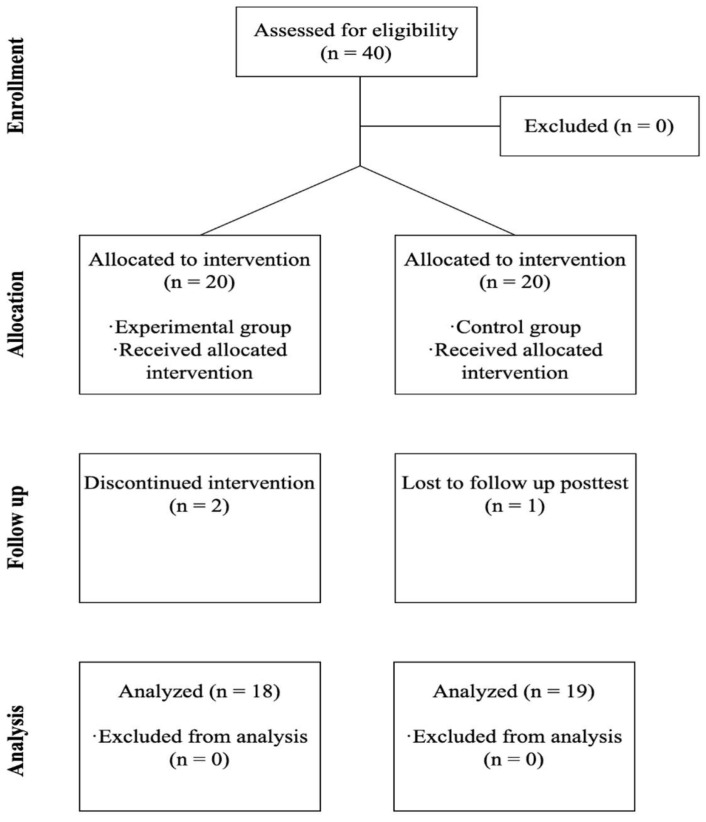
Flow of participants’ progress in this study.

The inclusion criteria were being third-year nursing students and being enrolled in the subject for the first time. The exclusion criterion was having had any experiences of any infection control education and FL. The third-year nursing students were selected based on the findings of previous research [6,23,24]. Additionally, these students had accumulated one year of clinical practicum, and it was anticipated that this education would prepare them to apply infection control practice during their upcoming clinical practicum in the following semester. A sample size estimation in the t-test, using a significance level (α) = 0.05, a power (1 − β) = 0.09, and a medium effect size (d) = 0.25 [19,20,21], was calculated. As a result, 18 participants were required per group. A total of 40 participants were recruited. No participants were excluded; however, during the intervention, two students withdrew from the intervention group discontinued intervention), and one student withdrew from control group (lost to follow-up). Figure 1 shows the participants’ progress in the study.

### 2.3. Process of This Study

This study was conducted from 22 December 2022 to 17 February 2023. The process of this study was: pretest, intervention, posttest 1 (post intervention), FGI, and posttest 2 (after 4 weeks). The experimental group received the infection control education program using FL based on the ARCS model, and the control group received a conventional lecture program. Both programs were conducted for 3 weeks (three times a week). The researchers conducted the study from intervention to FGI. To prevent bias, a research assistant, who was not involved in the study and was unaware of the assignment of participants to the experimental and control groups, administered the pretest, posttest 1, and posttest 2.

### 2.4. Quantitative Study Progress

#### 2.4.1. Interventions

The infection control education program, using FL based on the ARCS model, was implemented. The education duration in our study spanned a period of 3 weeks, with classes held three times a week during the winter vacation. This duration was determined based on previous research, regarding literature reviews of infection control education for nursing students [25,26], which reported a range of education durations, from single 2-h sessions to a minimum of 3 weeks. The contents of the program and the ARCS strategies were based on prior research [19,20,21,23,24,25,26,27] regarding infection control education programs applied to nursing students. The program consisted of three learning subjects. The week 1 learning subject was health-associated infection diseases that frequently occur in hospitals, such as catheter-associated urinary tract infections, central line-associated bloodstream infections, and ventilator-associated pneumonia. The week 2 learning subject was standard precautions and transmission-based precautions that frequently occur in hospitals. Lastly, the week 3 learning subject was creating infection control guidelines through the analysis of infection control diseases, using case-based learning. The program was conducted using FL and consisted of the pre-class, the in-class, and the post-class.

##### Pre-Class

The pre-class videos, related to the learning subjects, were made by the researcher. To enhance the learners’ focus and interest, musical accompaniments were added at the start and conclusion of the pre-class videos. Additionally, quizzes and assignments were present in the video to engage learners with the content and facilitate SDL. Furthermore, a learning management system (LMS) was established to effectively manage the pre-class activities, the infection control education program, and interactions between instructors and learners. A week prior to the in-class session, the pre-class video and learning materials were shared on the LMS to facilitate SDL. During the pre-class, activities such as summarization, assessment, and making questions were promoted to enhance SDL. Learners were also encouraged to engage in SDL using their preferred approaches. Recognizing the significance of learner–teacher interaction in the learning process [13,28], progress was periodically monitored through attendance rates, access times, access frequency, and learners’ progress rates.

##### In-Class

During the in-class, learners’ prior knowledge was assessed, and team activities were conducted. In the week 1 class, the activities were based on understanding health-associated infection disease guidelines and organizing related literature. The instructor provided an online resource where relevant literature could be accessed, and learners discussed how infection control was applied from guidelines and literatures. In week 2, learners designed a poster to educate patients and caregivers about standard precautions and transmission-based precautions. Healthcare-associated infection prevention guidelines recommend that hospital staff should explain standard precautions to patients, their families, and visitors [27]. Furthermore, the procedure for putting on and taking off the personal protective equipment (PPE), critical in transmission-based precautions, was demonstrated. This process, involving hand washing, sterile gloves, PPE, goggles, masks, and face shields, facilitated an understanding of the rationale, sequence, and principles of putting on and taking off PPE. Additionally, fluorescent lotion and ultraviolet lamps were used during the process to enable learners to promptly assess contamination levels and grasp the importance of correct PPE usage. Lastly, in week 3, learners created infection control guidelines for each different cases. In the prior study [29], the application of case-based learning into the FL enhanced problem-solving skills, SDL abilities, and self-efficacy. This approach facilitated the consolidation of infection control content. To conclude the in-class, learners reviewed the material by answering multiple-choice questions provided by the instructor and performed self-assessments on individual and team activities.

##### Post-Class

Following the class, learners engaged in keeping a reflective journal. It served to document the learning process and experiences. The learners shared their reflective journals through the LMS and received feedback. Table 1 represented an implementation of FL based on ARCS strategies.

#### 2.4.2. Experimental Group and Control Group

The experimental group received the infection control education program, using FL based on the ARCS model, for 3 weeks. The control group received a conventional lecture on infection control once a week for 3 weeks. The lecture materials contained the same content as provided to the experimental group. Moreover, the lectures were performed in person, utilizing both PowerPoint presentations and a blackboard during face-to-face sessions.

#### 2.4.3. Measurements

##### Learning Motivation

Learning motivation was measured using a learning motivation subscale [30] based on Keller’s questionnaire [31]. There were 27 items rated on a five-point Likert scale, ranging from “strongly disagree” (1) to “strongly agree” (5), with higher scores indicating higher learning motivation. The Cronbach’s α in this study was 0.93.

##### Self-Directed Learning Ability

SDL ability was measured using the SDL readiness scale [32] developed by West and Bentley [33]. There were 32 items rated on a five-point Likert scale, ranging from “strongly disagree” (1) to “always” (5), with higher scores indicating higher SDL ability. The Cronbach’s α in this study was 0.92.

##### Academic Self-Efficacy

Academic self-efficacy was measured using an academic self-efficacy tool [34] developed by Ayres [35]. There were 10 items rated on a seven-point Likert scale, ranging from “strongly disagree” (1) to “strongly agree” (7), with higher scores indicating higher academic self-efficacy. The Cronbach’s α in this study was 0.93.

##### Knowledge and Confidence in Infection Control Practice

Knowledge on and confidence in infection control practice were measured using the researchers’ development tool. Items were developed based on the guidelines for Standard precautions for healthcare-associated infections [27] and guidelines for isolation precautions in healthcare settings [36]. First, 40 preliminary items were extracted from the guidelines. To verify the content validity index (CVI), it was identified by seven experts. Finally, 24 items with a CVI of 0.8 or higher were selected. The measurement was a true or false, rated using 1 point for “true” and 0 points for “false”, with a higher score indicating higher knowledge of infection control. The Kuder–Richardson Formula 20 in this study was 0.62.

Confidence in infection control practice was measured by modifying the predicate to ‘-can’ and ‘explain’ from the knowledge of infection control variable of this study. The measurements were rated on a five-point Likert scale, ranging from “very poor” (1) to “very good” (5), with higher scores indicating higher confidence in infection control practice. The Cronbach’s α in this study was 0.98.

##### Anxiety

Anxiety was measured using a standardized Korean version of Spielberger’s State-Trait Anxiety Inventory [37] by Kim and Shin [38]. There were 20 items, rated on a four-point Likert scale, ranging from “strongly disagree” (1) to “strongly agree” (4), with lower scores indicating lower anxiety. The Cronbach’s α in this study was 0.91.

### 2.5. Qualitative Study Progress

After the program’s completion, FGI was administered to eight participants from the experimental group, all of whom had participated in the full three-session program. The FGI sessions were organized by dividing the participants into four groups, each containing two participants. Each interview session lasted approximately 20–30 min. The researcher facilitated FGI in a discrete place, selected to honor participant privacy and preserve the natural progression of narratives. In cases where face-to-face interviews were impossible, interviews were conducted using a video conferencing platform (Zoom). In the pursuit of bolstering the qualitative research validity, consideration was given to the principles of reliability, auditability, and confirmability [39]. First, credibility was upheld by minimizing potential researcher biases and emotional influences. An atmosphere in which participants could openly and deeply articulate their experiences was cultivated, facilitating an authentic portrayal of their perspectives. Second, to augment the study’s reliability and ensure auditability, detailed records were meticulously maintained, with interview specifics such as dates, locations, times, and participant reflections. During the subsequent data analysis phase, two researchers independently assessed the data and engaged in continuous discourse to deliberate their individual analyses. This sustained dialogue culminated in a convergence of interpretations, bolstering the integrity of the analysis process. Lastly, for confirmability, rigorous efforts were exerted to derive conclusions solely from the data obtained through the interview process. This approach highlighted the results solely from the practical knowledge obtained from the interviews.

### 2.6. Data Analysis

#### 2.6.1. Quantitative Analysis

The collected data were analyzed using the SPSS version 25.0 program. First, the demographic and the study variables were analyzed using frequency/percentage and mean/standard deviation. A Shapiro–Wilk test was conducted to test the normality of the study variables. A Chi-squared test with Fisher’s exact test on the study variables, or an independent t-test on the study variables, was conducted to test homogeneity. Second, the effects of infection control education, using FL based on the ARCS model, were analyzed using a linear mixed-effects model to compare the interaction differences between three time points, before and after completing the interventions.

#### 2.6.2. Qualitative Analysis

The qualitative data were analyzed using conventional content analysis [40] to explore the experiences of participants. The researchers thoroughly reviewed the transcribed data, immersing themselves in its content to grasp its overall essence. Subsequently, they meticulously examined the data word by word to identify codes that effectively represented the participants’ experiences of infection control education using FL based on the ARCS model. Additionally, the researchers recorded their initial thoughts and feelings during this process and continued to analyze the data to develop relevant codes. These codes were further organized into subthemes based on their relationships and connections with other codes. Finally, overarching themes were derived by grouping subthemes according to their thematic connections. Throughout the data analysis process, the researchers held multiple meetings for ongoing communication, discussion, and consensus-building. The researchers also regularly revisited and revised the identified codes, subthemes, and themes in the dataset.

### 2.7. Ethical Consideration

This study was approved by the GNU Institutional Review Board (IRB No: GIRB-A22-Y-0072). Participation was voluntary, anonymity and confidentiality were ensured, and participants could withdraw at any time. Written informed consent was obtained. For qualitative data, explicit consent was obtained to record interviews. Individualized IDs were used to protect participant identities during computer-based transcription, ensuring confidentiality.

## 3. Results

### 3.1. Quantitative Results

#### 3.1.1. Homogeneity Test of General Characteristics and Variables in Pretest

There was no statistically significant difference between the experimental group and the control group in the general characteristics. Table 2 represents the general characteristics and the homogeneity test.

#### 3.1.2. Effects of the Infection Control Education Program Using Flipped Learning Based on the ARCS Model

**Figure 2:**
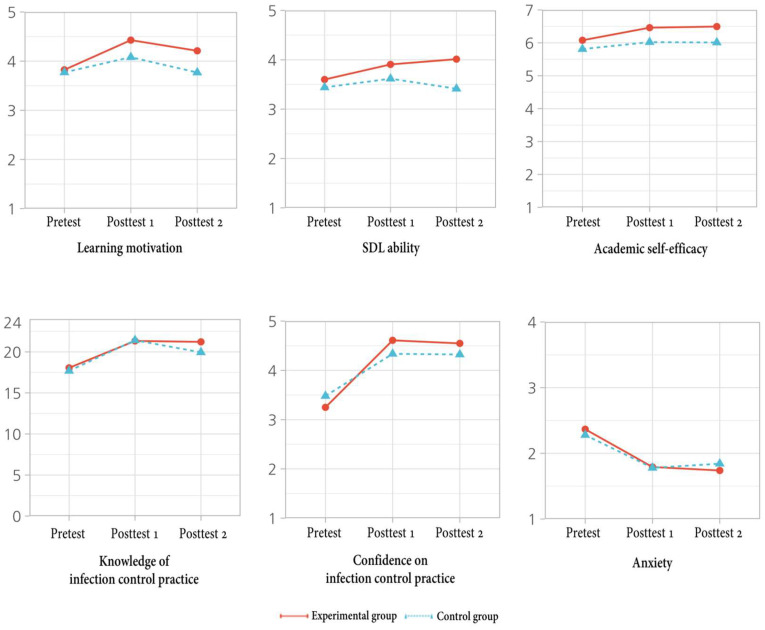
Time series of variables between experimental group and control group.

There were statistically significant interaction differences between the group and time in learning motivation (t = 2.29, *p* = 0.025), SDL ability (t = 2.69, *p* = 0.009), and confidence in infection control practice (t = 2.41, *p* = 0.009). There were no statistically significant interaction differences between the group and time in academic self-efficacy (t = 1.11, *p* = 0.272), knowledge of infection control (t = 0.74, *p* = 0.459), and anxiety (t = −1.23, *p* = 0.224). Table 3 represents the results of the linear mixed-effects model for variables and Figure 2 indicates the time series of variables between experimental group and control group.

### 3.2. Qualitative Results

In the results of the focus group interview, the derived themes were ‘Studying with learning motivation’, ‘Self-directed studying’, ‘Interesting learning infection control’, and ‘Difficulty in new ways of learning’. The major theme was ‘Engaging learning experience, although difficult, in new ways of learning’.

#### 3.2.1. Theme 1: Studying with Learning Motivation

The participants reported that during the pre-class, they were able to grasp the core concepts and flow of the learning contents. They also mentioned that they could engage better in learning by maintaining attention while solving problems or answering questions. Additionally, they expressed that their understanding of infection control improved through the use of real cases and easily understandable examples. Also, they highlighted that they gained confidence in learning.

*“In the pre-class,* *I got really curious with the introduction of gaps, so I ended up diving deep into the material.”*

*(Participant 4)*

*“The instructor presented real cases and examples aiding my understanding and attention. Learning about infection control through videos and images made it easier to grasp.”*

*(Participant 7)*

*“Having prepared before class, I actively participated and developed the confidence to engage.”*

*(Participant 3)*

#### 3.2.2. Theme 2: Self-Directed Learning

The participants expressed that they cultivated a proactive attitude by setting learning goals and assessing their learning progress. They stated they had the ability to study independently at their preferred pace, using their own methods.

*“Setting my own learning goals was a memorable experience. This self-set target motivated me to work harder. And post-class reflection enabled me to identify areas where I lacked and prompted me to address those gaps.”*

*(Participant 6)*

*“Filling out a reflection helped me evaluate my progress rather than merely completing the class. I focused on improving my weak points.”*

*(Participant 8)*

*“I went over what I learned and asked questions while studying on my own, trying to find more ways to get better.”*

*(Participant 4)*

#### 3.2.3. Theme 3: Interesting Learning Infection Control

The participants discovered that the process of understanding infection control concepts was interesting. Moreover, they mentioned that they comprehended the necessity of infection control, the associated methods, and why practicing it is important.

*“I really got the idea of infection control from the interesting class.”*

*(Participant 1)*

*“Understanding why we need infection control and how it’s done was really helpful. The class was enjoyable, and that really helped comprehend the concept of infection control.”*

*(Participant 3)*

#### 3.2.4. Theme 4: ‘Difficulty in New Ways of Learning’

Participants found the pre-class flexible but sometimes burdensome, due to the time investment required. They also mentioned the challenges posed by the content of the pre-class assignments suggested by the instructor.

*“Even though it seemed easy to just watch those videos before class, it actually required more time, which kind of made it a bit harder. And also, some difficulties during team activity when the pre-class didn’t do actively.”*

*(Participant 2)*

## 4. Discussion

This program has shown the efficacy of the FL method, especially when based on the ARCS model. Considering the limited application of FL on the ARCS model in the realm of infection control education, it becomes imperative to contemplate the incorporation of FL and the ARCS model in infection control education.

As specific effects, learning motivation increased significantly in the experimental group compared to the control group. Similar positive changes were reported in studies documenting the application of the ARCS model in instructional contexts [17,19,20,21,41,42]. As a means of enhancing learning motivation, a range of infection control-related materials were presented to capture perceptual attention based on the previous studies [17,19,20,21]. Contents were offered in the form of quizzes to foster an inquisitive mindset among learners [19,20]. Notably, extensive attention was paid to the pre-class in this study. Learners set personalized learning goals, were provided with literature, examples, and quizzes, and were encouraged to pose questions during pre-videos to stimulate active thought processes [17,19,20,21]. Also, as Keller [17] mentioned, the strategy that consistently underscored the advantages of infection control, its real-life and clinical field applications, and its practical relevance, led to increases in learners’ learning motivation. In addition, as a strategy to increase the confidence of the learners, the level of challenge was considered, by providing cooperative learning that applied the learning contents from the clear and easy tasks in the pre-class to the in-class sessions. It showed that the learning motivation of the learners was improved, through the strategies to choose and solve problems by themselves during cooperative learning. These strategies were applied so that learners could evaluate the learning process and express a sense of achievement through reflection in the post-class. In this study, it is meaningful that the learning motivation of the learners was further strengthened by applying the ARCS strategy step-by-step to the process of the in-class and the post-class.

The SDL ability was statistically significantly increased in the experimental group compared to the control group. Similar to previous studies [20,43], this study also confirmed that FL is an effective learning method for improving SDL ability. Particularly, the results suggest that the improvement in learning motivation in this study influenced the increase in SDL ability. It emphasized the motivational aspect of SDL [15], and learning motivation is an important factor in SDL ability. Therefore, in future research, it is necessary to verify the effect on SDL ability by implementing FL, based on the ARCS model, in different educational topics.

Academic self-efficacy had no significant difference between the two groups. A previous study [44] that applied the ARCS model also showed no effect on self-efficacy, which was similar to the results of this study. Because self-efficacy develops gradually through repeated learning experiences, a sufficient learning period is required [45]. Therefore, although this study was based on the ARCS model, it is likely that the period was not sufficient to sustain academic self-efficacy, so a follow-up study with a longer period is needed to verify this.

Knowledge of infection control showed no significant difference between the two groups. Although ARCS strategies were applied in this study to improve knowledge, it is likely that knowledge increased because the control group in this study also focused on the delivery and understanding of learning contents through conventional lectures. In the previous study [16], when the ARCS model was applied, the effect on academic achievement was found to be inconsistent, supporting the results of this study. In this study, the knowledge of infection control was measured by researchers’ development tool, which consisted of true or false questions. Lee [12] emphasized designing classes so that the restructuring of learned knowledge, or the in-depth learning of knowledge, can occur while considering the level of the learners, when designing the FL. An evaluation method that confirms reliability and objectivity should be sought, so that evaluation can be conducted, centered on the process rather than simply measuring the improvement in knowledge.

Confidence in infection control practice was significantly different between the two groups. The increase in confidence in infection control practice can be attributed to the execution of challenging learning tasks, accompanied by a sense of satisfaction experienced during the learning. However, based on the correlation between confidence in practice and practice [46], it was expected that performance would increase as confidence in infection control practice increased. Since infection control practice was not actually observed in this study, it is necessary to measure infection control practice in future studies.

There was no significant difference in anxiety between the two groups. These results were similar to the absence of an effect on anxiety in the ARCS applied group [47]. As mentioned by Keller [17], ARCS strategies were implemented to bolster learners’ confidence as a means of managing learner anxiety in this study. However, it did not significantly reduce the learners’ anxiety. Also, learners experienced difficulties in new ways of learning and had some difficulty understanding through the experiences of participation.

The effects of the infection control education program, using FL based on the ARCS model, were increased learning motivation, SDL ability, and confidence in infection control practice, but there were no effects on academic self-efficacy, knowledge of infection control, and anxiety. In addition, in the qualitative results, ‘difficulty in new ways of learning’ was found. In future research, education programs based on the ARCS model that can increase the learners’ learning motivation should be conducted.

The limitations of this study are as follows: first, this study did not directly measure infection control practice. Therefore, it is advisable to implement the education either before or during the clinical practicum, ensuring a seamless transition to practice. Second, although this study implemented an education program based on the ARCS model, it showed no significant effects on self-efficacy, knowledge, and anxiety. Thus, strategies aimed at further enhancing learning motivation are warranted. Third, it is essential to implement effective ARCS strategies at each stage of flipped learning. Particularly, the pre-class stage is where SDL occurs, so strategies that can demonstrate an impact on SDL ability should be reinforced. Finally, we emphasize the potential benefits of a three-week educational intervention in enhancing learning motivation and SDL ability. However, three weeks is a relatively short duration, and to assess the long-term impact and sustainability of this education, future research with a longer timeframe will be necessary.

## 5. Conclusions

This study demonstrated that the experimental group, receiving an infection control education program using FL based on the ARCS model, had shown significant improvements in learning motivation, SDL ability, and confidence in infection control practice.

Based on the results of this study, there are some implications. First, given the demonstrated effects of the infection control education program for nursing students in this study, its application can be extended to nursing practice. Especially, it could provide an essential component of infection control education for both nursing students and new nurses. Second, because these pedagogical approaches significantly enhanced SDL ability by increasing learning motivation, similar educational programs could be considered for nursing students on various subjects, aimed at further cultivating SDL abilities.

## Figures and Tables

**Table 1:** Infection control education program using flipped learning based on the ARCS model.

Learning Subject	Flipped Learning	ARCS Strategies
**Week 1** Health-associated infection diseases **Weeks 2** Standard precautions and transmission-based precautions **Weeks 3** Case study of infection control	**Pre-class** Watching a series of pre-recorded videos for learning subjects (15–20 min per video). Self-directed and asynchronous learning Setting learning objectives Summarizing pre-videos in notes Researching related cases or literature and summarizing Formulating questions Completing quizzes **In-class** Discussing questions and feedback Doing team activities Searching guidelines Making posters related to standard precautions and transmission-based precautions Case studies of various scenarios Presenting findings from team activity Mini lecture to recall Completing multiple-choice quizzes **Post class** Formulating the reflective journal Sharing their reflective journal Assessing their achievement of set learning objectives Receiving feedback from instructor	**Attention** Use multimedia materials such as voice, text, picture, and video for learning contents Provide related videos for motivation at every session, curiosity through pre-quiz Provide paper for summarizing, Make learners complete pre-quiz and create questions for an effective pre-class Empower learners to establish their own learning objectives. Diversify teaching methods, including video-based and written materials for instruction **Relevance** Emphasize that infection control is a fundamental and essential practice in clinical settings Explain the benefits of infection control Communicate that learners can actively engage in infection control practice and contribute to infection prevention Providing contents consistent with learning goals while showing cases of infection control in clinical practice Provide activity tasks based on the knowledge, experience, and information known to learners Explain concepts using precise terminology, accompanied by illustrative visuals and videos **Confidence** Foster learner expectations for the learning process by providing guidance on content and tasks Present challenges and opportunities for success through quizzes and team activities in each class Enable learners to choose their tasks, promoting a sense of autonomy Structure learning content by linking it to clinical practice experiences **Satisfaction** Provide positive feedback on learners’ learning efforts Evaluate the learning achievement through self-reflection and self-assessment

**Table 2:** General characteristics and homogeneity test between the experimental group and control group (N = 37).

Variables	Categories	Exp. (*n* = 18)	Cont. (*n* = 19)	χ^2^ or t	*p*
		*n* (%)	*n* (%)		
Age (years)		23.22 ± 2.60	25.37 ± 7.36	−1.19	0.244
Sex	Male	1 (5.6)	2 (10.5)	-	0.100 ^†^
	Female	17 (94.4)	17 (89.5)		
Period of clinical practice (weeks)		6.00 ± 0.00	5.89 ± 0.32	1.46	0.163
Major satisfaction	Unsatisfied	0 (0.0)	1 (5.2)	1.08	0.100 ^†^
	Moderate	5 (27.8)	6 (31.6)		
	Satisfied	13 (72.2)	12 (63.2)		
College life satisfaction	Unsatisfied	0 (0.0)	0 (0.0)	-	0.062 ^†^
	Moderate	2 (11.1)	8 (42.1)		
	Satisfied	16 (88.9)	11 (57.9)		
Clinical practice satisfaction	Unsatisfied	0 (0.0)	0 (0.0)	-	0.151 ^†^
	Moderate	3 (16.7)	8 (42.1)		
	Satisfied	15 (83.3)	11 (57.9)		
Experience of exposure to infection diseases	Yes	3 (16.7)	4 (21.1)	-	0.100 ^†^
	No	15 (83.3)	15 (78.9)		
Experience of COVID-19	Yes	14 (77.8)	13 (68.4)	-	0.714 ^†^
	No	4 (22.2)	6 (31.6)		
		Mean ± SD		t	*p*
Learning motivation		3.83 ± 0.27	3.78 ± 0.33	0.46	0.651
SDL ability		3.60 ± 0.33	3.44 ± 0.29	1.55	0.13
Academic self-efficacy		6.08 ± 0.76	5.81 ± 0.23	1.43	0.167
Knowledge of infection control practice		18.06 ± 2.58	17.68 ± 2.83	0.42	0.68
Confidence in infection control practice		3.25 ± 0.46	3.48 ± 0.37	−1.67	0.103
Anxiety		2.37 ± 0.15	2.28 ± 0.24	1.34	0.189

^†^: Fisher’s exact test; Exp = experimental group; Cont = control group; SDL = self-directed learning.

**Table 3:** Results of linear mixed-effects model for variables (N = 37).

Variables	Source	Estimate	SE	t	*p*	95% CI	
						Lower	Upper
Learning motivation	Intercept	3.89	0.14	28.05	<0.001	3.61	4.16
	Group	−0.12	0.2	−0.58	0.56	−0.51	0.28
	Time	−0.01	0.06	−0.01	0.923	−0.13	0.11
	Group × Time	0.2	0.08	2.29	0.025	0.03	0.37
SDL ability	Intercept	3.52	0.13	27.22	<0.001	3.26	3.77
	Group	−0.09	0.19	−0.50	0.622	−0.46	0.28
	Time	−0.01	0.06	−0.23	0.818	−0.13	0.1
	Group × Time	0.22	0.08	2.69	0.009	0.06	0.38
Academic self-efficacy	Intercept	5.75	0.16	36.59	<0.001	5.44	6.06
	Group	0.18	0.23	0.8	0.425	−0.27	0.63
	Time	0.1	0.07	1.46	0.148	−0.04	0.24
	Group × Time	0.11	0.1	1.11	0.272	−0.09	0.3
Knowledge of infection control practice	Intercept	17.42	0.93	18.75	<0.001	15.58	19.26
	Group	−0.38	1.33	−0.29	0.774	−3.03	2.26
	Time	1.13	0.42	2.67	0.009	0.29	1.98
	Group × Time	0.45	0.61	0.74	0.459	−0.76	1.66
Confidence in infection control practice	Intercept	3.24	0.14	22.37	<0.001	2.95	3.53
	Group	−0.34	0.21	−1.63	0.111	−0.76	0.08
	Time	0.42	0.07	6.43	<0.001	0.29	0.56
	Group × Time	0.23	0.09	2.41	0.022	0.03	0.42
Anxiety	Intercept	2.41	0.13	17.95	<0.001	2.14	2.67
	Group	0.19	0.19	0.99	0.325	−0.19	0.57
	Time	−0.22	0.54	−4.02	<0.001	−0.33	−0.11
	Group × Time	−0.10	0.08	−1.23	0.224	−0.25	0.06

SE = standard error; CI = confidence interval.

## Data Availability

The data presented in this study are available from the corresponding author upon reasonable request.
